# Third-Generation Cephalosporin-Loaded Chitosan Used to Limit Microorganisms Resistance

**DOI:** 10.3390/ma13214792

**Published:** 2020-10-27

**Authors:** Letiția Doina Duceac, Gabriela Calin, Lucian Eva, Constantin Marcu, Elena Roxana Bogdan Goroftei, Marius Gabriel Dabija, Geta Mitrea, Alina Costina Luca, Elena Hanganu, Cristian Gutu, Liviu Stafie, Elena Ariela Banu, Carmen Grierosu, Alin Constantin Iordache

**Affiliations:** 1 Faculty of Dental Medicine, “Apollonia” University of Iasi, 11 Pacurari Str., 700511 Iasi, Romania; letimedr@yahoo.com (L.D.D.); dr_liviustafie@yahoo.com (L.S.); grierosucarmen@yahoo.com (C.G.); 2 Nicolae Oblu Neurosurgery Hospital of Iasi, 2 Ateneului, 700309 Iasi, Romania; mariusdabija.md@gmail.com (M.G.D.); alinciordache@gmail.com (A.C.I.); 3 Faculty of Medicine and Pharmacy, University Dunarea de Jos, 47 Domneasca Str., 800008 Galati, Romania getamitrea@yahoo.com (G.M.); dr_cgutu@gmail.com (C.G.); banuariela@yahoo.com (E.A.B.); 4 Saarbrucken-Caritasklink St.Theresia University Hospital, 66113 Saarbrücken, Germany; 5 Sf. Ioan, Emergency Clinical Hospital, 2 Gheorghe Asachi Str., 800494 Galati, Romania; 6 “Grigore T. Popa”, University of Medicine and Pharmacy of Iasi, 16 Universitatii Str., 700115 Iasi, Romania; acluca@yahoo.com (A.C.L.); dr.elenahanganu@gmail.com (E.H.); 7 Sf. Ap. Andrei Emergency Clinical Hospital, 177 Brailei Str., 800578 Galati, Romania; 8 Sf. Maria Emergency Clinical Hospital for Children of Iasi, 62 Vasile Lupu Str., 700309 Iasi, Romania; 9 Discipline of Pediatric Surgery and Orthopedics, Faculty of Medicine, “Grigore T. Popa” University of Medicine and Pharmacy of Iasi, 16 Universitatii Str., 700115 Iasi, Romania; 10 Emergency Military Hospital, 199 Traian Str., 800150 Galati, Romania; 11 Public Health Directorate of Iasi, 2-4 Vasile Conta, 7001016 Iasi, Romania; 12 Orthopaedic Trauma Surgery Clinic, Clinical Rehabilitation Hospital, 14 Pantelimon Halipa Str., 700661 Iasi, Romania

**Keywords:** chitosan, cardiology, pediatrics, epidemiology, neurosurgery, pediatric surgery, pulmonology, obstetrics/gynecology, orthopedics

## Abstract

From their discovery, antibiotics have significantly improved clinical treatments of infections, thus leading to diminishing morbidity and mortality in critical care patients, as well as surgical, transplant and other types of medical procedures. In contemporary medicine, a significant debate regarding the development of multi-drug resistance involves all types of pathogens, especially in acute care hospitals due to suboptimal or inappropriate therapy. The possibility of nanotechnology using nanoparticles as matrices to encapsulate a lot of active molecules should increase drug efficacy, limit adverse effects and be an alternative helping to combat antibiotic resistance. The major aim of this study was to obtain and to analyze physico-chemical features of chitosan used as a drug-delivery system in order to stop the antibiotic resistance of different pathogens. It is well known that World Health Organization stated that multidrug resistance is one of the most important health threats worldwide. In last few years, nano-medicine emerged as an improved therapy to combat antibiotic-resistant infections agents. This work relies on enhancement of the antimicrobial efficiency of ceftriaxone against gram(+) and gram(−) bacteria by antibiotic encapsulation into chitosan nanoparticles. Physicochemical features of ceftriaxone-loaded polymer nanoparticles were investigated by particle size distribution and zeta potential, Fourier-transform infrared spectroscopy (FTIR), Thermal Gravimetric Analysis (TG/TGA), Scanning Electron Microscopy (SEM) characteristics techniques. The obtained results revealed an average particle size of 250 nm and a zeta potential value of 38.5 mV. The release profile indicates an incipient drug deliverance of almost 15%, after 2 h of approximately 83%, followed by a slowed drug release up to 24 h. Characteristics peaks of chitosan were confirmed by FTIR spectra indicating a similar structure in the case of ceftriaxone-loaded chitosan nanoparticles. A good encapsulation of the antibiotic into chitosan nanoparticles was also provided by thermo-gravimetric analysis. Morphological characteristics shown by SEM micrographs exhibit spherical nanoparticles of 30–250 nm in size with agglomerated architectures. Chitosan, a natural polymer which is used to load different drugs, provides sustained and prolonged release of antibiotics at a specific target by possessing antimicrobial activity against gram(+) and gram(−) bacteria. In this research, ceftriaxone-loaded chitosan nanoparticles were investigated as a carrier in antibiotic delivery.

## 1. Introduction

Nowadays, many researchers are focused on the evolution mechanism of microbial resistance to broad-spectrum antibiotics in order to develop novel formulations of antimicrobial agents that reveal increased activity against serious multidrug-resistant pathogen agents. Reduced bacteria cellular penetration limits the efficiency of many antibiotic current therapies, thus decreasing their activity against various microbial infections. It is obvious that bacteria have adapted survival mechanisms over time, which allow them to occur in any environmental condition, representing an important challenge to the medical field and healthcare procedures [1,2]. Strategies on infection management take the prolonged contact time of antibiotics with pathogens into account, and so identified antimicrobial materials based on polymers. Therefore, chitosan, a natural polysaccharide, is a highly biocompatible nanomaterial possessing antimicrobial activity, non-toxicity, non-antigenicity and biodegradability. The mechanism of its action consists of connection to the bacterial cell wall followed by alteration in the cell membrane and reduced permeability, and ending with binding to bacteria deoxyribonucleic acid (DNA), thereby inhibiting its replication. It has been demonstrated that chitosan acts as an antimicrobial agent against both gram (−) and gram (+) bacteria. Considering all these issues, drug potency against pathogens could be enhanced by loading chitosan nanoparticles with active biomolecules, inducing synergistic action against microorganisms [3,4,5,6,7,8]. Ceftriaxone is a broad-spectrum, third-generation cephalosporin, launched in 1988 in the community for several infections’ treatment or multi-resistant strain infections. It is a β-lactamase-resistant antibiotic with an exceptional serum half-life, up to ten times longer than other antibiotics belonging to this class. Ceftriaxone is administered to newborns, children and adults to treat various infections such as bacterial meningitis, acquired community pneumonia, hospital-acquired pneumonia, acute media otitis, intra-abdominal infections, complicated urinary tract infections (including pyelonephritis), gonorrhea, pox, infections of bones and joints, complicated skin and soft tissue infections, bacterial endocarditis, for the treatment of acute exacerbations of chronic obstructive pulmonary disease in adults, for the treatment of disseminated Lyme borreliosis (early stage and advanced disease stage) in adults and children, including infants over 15 days of age, for preoperative prophylaxis of local infections associated with surgery, in the control of neutropenia in patients with fever, which is suspected to be caused by a bacterial infection and for the treatment of patients with bacteremia associated with, or suspected of being associated with, any of the infections mentioned above. For this reason, ceftriaxone is used in various medical fields such as cardiology, pediatrics, epidemiology, neurosurgery, pediatric surgery, pulmonology, obstetrics/gynecology, orthopedics [9,10,11,12,13,14].

**Figure 1:**
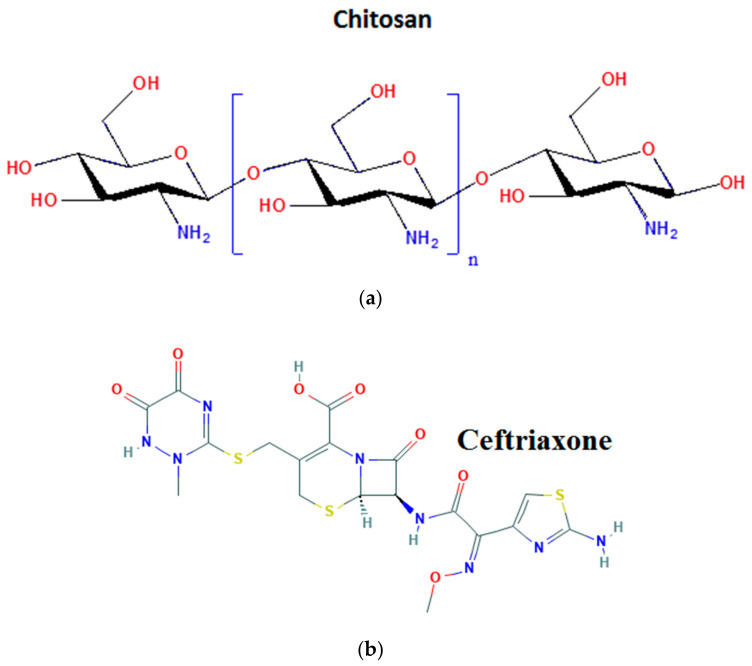
Schematic representation of chitosan (**a**) and ceftriaxone (**b**).

Figure 1 presents a schematic view of chitosan and ceftriaxone. An encouraging strategy to ensure efficacy in drug therapy is the evolution of suitable drug delivery systems. There are studies reporting the use of polymer nanoparticles as a self-assembled vehicle to deliver the drug to the target site due to the unique properties such as biodegradability, biocompatibility, antimicrobial qualities and non-toxicity [15,16,17]. Their capacity to incorporate various active molecules offers the possibility of enhancing the bactericidal and bacteriostatic efficacy of the antibiotic, limiting, at the same time, the adverse effect [18,19,20].

In order to limit ceftriaxone resistance, drug carriers based on chitosan nanoparticles may increase the therapeutic efficiency of the antibiotic by prolonging its release as well as minimizing its side effects [21,22,23,24,25,26,27].

These delivery systems improve the usage of novel formulations by perfecting preparation methods and bringing together natural or synthetic polymer nanoparticles, which were extensively engaged in pharmaceutical and medical areas, and active molecules used in different treatments. Thereby, these approaches include a wide variety of anti-infection agents as, currently, the medical field worldwide is confronted with multidrug-resistant microorganisms. The prolonged and controlled release of the encapsulated drug consists of its higher concentration in the circulatory system, which obtains the average concentration by keeping away the side effects.

In this study, we prepared ceftriaxone-loaded chitosan nanoparticles and analyzed their physico-chemical features.

## 2. Materials and Methods 

### 2.1. Material Synthesis

**Figure 2:**
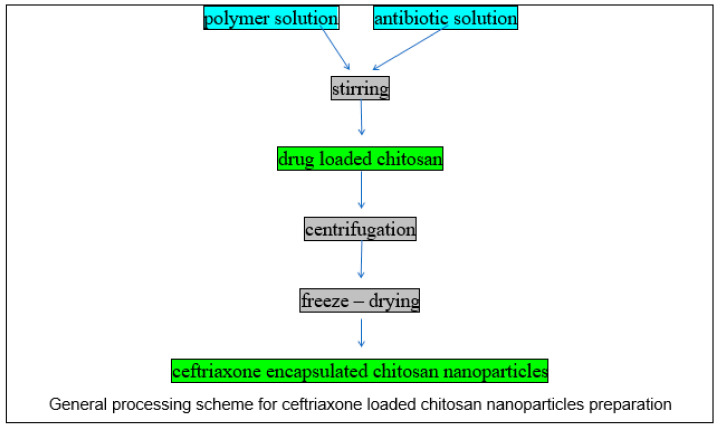
General processing scheme for ceftriaxone-loaded chitosan nanoparticles preparation.

Samples containing ceftriaxone-intercalated chitosan nanoparticles were obtained using chitosan as a polymer and trisodium-polyphosphate (TPP) as a crosslinking agent by the coacervation method. A pre-established amount of chitosan was dissolved in 10 mL acetic acid (1% *v*/*v*) and the pH of the solution was maintained at 5.0 by adding 1 M NaOH solution under sonication at room temperature. The degree of deacetylation of the chitosan is 80.3% according to the producer’s certificate. Three amounts of ceftriaxone, 25, 50 and 100 mg, were dissolved in ultrapure water before being added to the as-prepared polymer and continuously stirred for 60 min. Then, 1 mg/mL TPP was added to the antibiotic–polymer solution under stirring at 800 rpm for 5 h in order to obtain ceftriaxone-loaded chitosan nanoparticles. Finally, the fresh prepared nanostructures were centrifuged and freeze-dried for 24 h (Figure 2). 

The obtained nano-scaled formulations were stored at 5 °C and further analyzed.

### 2.2. Characterization Methods

Simultaneous thermal analysis STA 449 F1 Jupiter by NETZSCH (Selb, Germany) was used to investigate the compositional analysis of multi-component materials or blends; thermal stabilities; oxidative stabilities; estimation of product lifetimes; decomposition kinetics; effects of reactive atmospheres on materials; filler content of materials; moisture and volatile content.

Particle size distribution was evaluated using a ZetaSizer Nano ZS analyzer (Malvern, UK), for the measurements of the particles size in the range of 1–8000 nm (by dynamic light scattering at an angle of 90°, using a He-Ne laser at λ = 633 nm) and zeta potential of the nanoparticles. 

Scanning Electron Microscope (Thermo Fisher Scientific, Waltham, MA, USA) equipped with an energy dispersive spectrometer (EDS, EDAX Octane Elite) allows for complete, high-resolution morphological investigations of this type of material.

FTIR spectrometer, Model Vertex 70 by Bruker (Berlin, Germany) was used to determine the structure and molecular composition of the samples in spectral domain: mid IR (5000 ÷ 400 cm^−1^), far IR (400 ÷ 50 cm^−1^).

## 3. Results

Chitosan nanoparticles were prepared by the ion gelation method, and the loading process of ceftriax-one-chitosan nanoparticles was accomplished by varying the concentration of antibiotic active molecule, as shown in Table 1.

**Figure 3:**
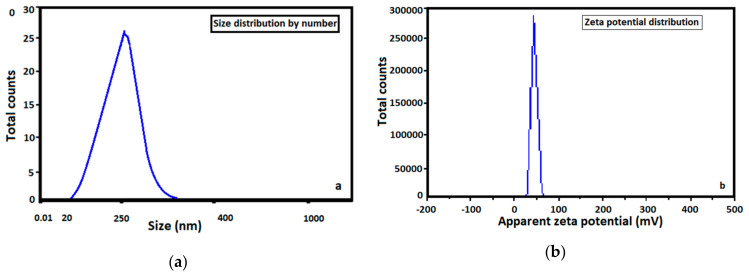
(**a**) Particle size distribution of ceftriaxone-loaded chitosan nanoparticles and (**b**) zeta potential of ceftriaxone loaded chitosan nanoparticles.

Particle size distribution and zeta potential are two parameters considered important in drug delivery (Figure 3). Ceftriaxone-encapsulated chitosan nanoparticles reveal an average particle size of 250 nm and a zeta potential value of 38.5 mV, indicating the stability of nanoparticles and high electric surface charge on antibiotic-loaded chitosan nanoparticles.

**Figure 4:**
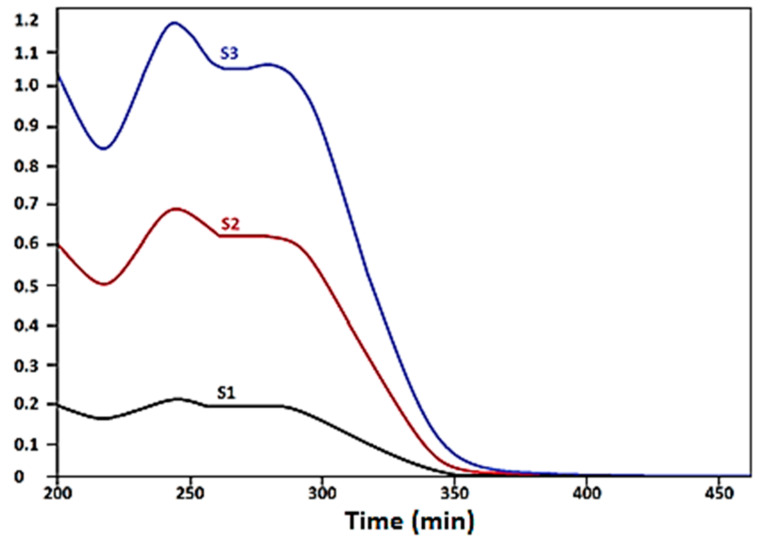
UV–VIS spectrum of S1, S2, S3.

**Figure 5:**
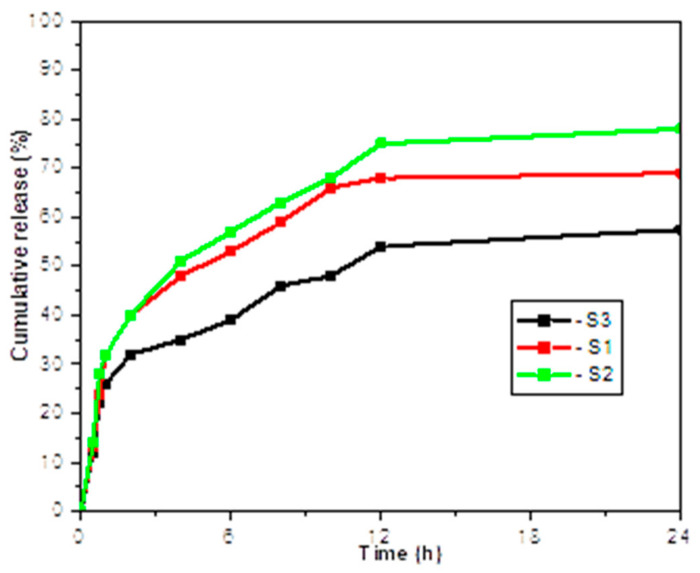
In vitro drug release profile of ceftriaxone-loaded chitosan nanoparticles.

The drug release profile (Figure 4 and Figure 5) was performed at a pH value of 7.4 and 37 °C and occurred by several operations such as surface erosion, disintegration, diffusion and desorption. There was a continual mixing indicating a rapid release of ~20% over the first 2 h, a steadier release from 2 to 12 h and from 12 to 24 h there were minor ceftriaxone releases of from 5 to 10% for each complex.

**Figure 6:**
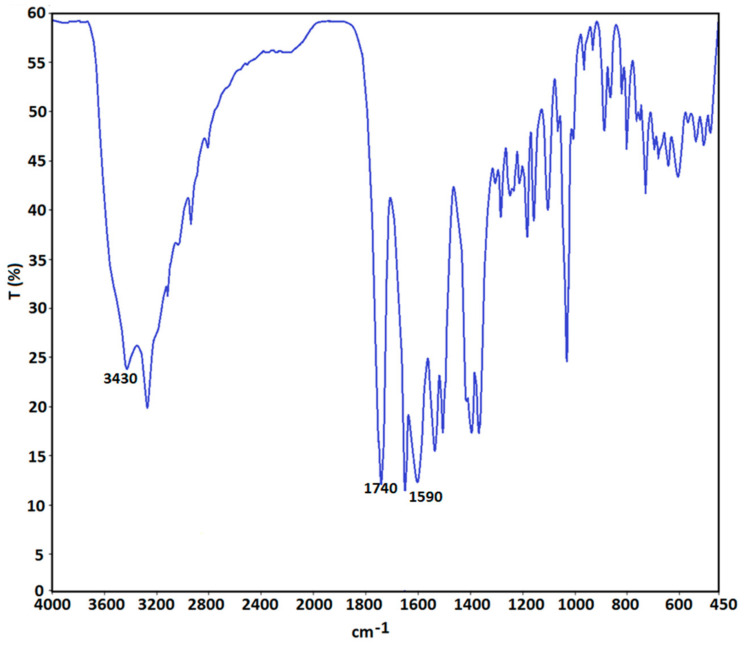
FTIR spectrum of ceftriaxone loaded chitosan nanoparticles.

The FTIR spectrum of ceftriaxone encapsulated chitosan nanoparticles presented in Figure 6 revealed characteristic peaks at around 3430 cm^−1^ of the N-H stretching vibration, at around 1740 cm^−1^ for the C=O stretching vibration and at 1590 cm^−1^ for the C=N stretching vibration, indicating that IR spectra matches with antibiotic spectra.

Furthermore, characteristic peaks of chitosan were noticed, and there was no modification of characteristic peaks, denoting the lack of chemical interaction between ceftriaxone and chitosan nanoparticles.

**Figure 7:**
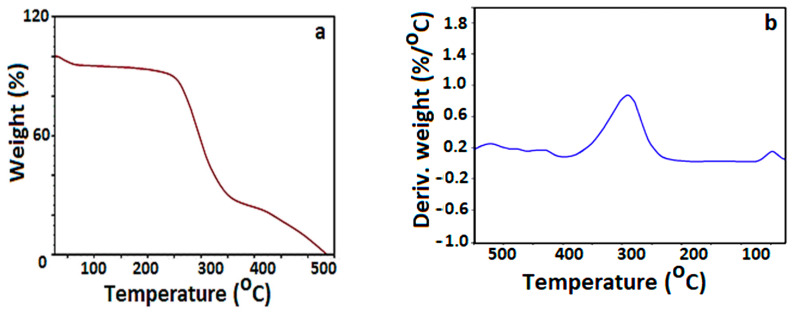
Thermo-gravimetric analysis of ceftriaxone encapsulated chitosan nanoparticles: (**a**) TG; (**b**) DTA.

Thermo-gravimetric analysis (TGA) is used to evaluate the thermal stability of ceftriaxone-loaded chitosan nanoparticles and weight loss at various temperatures, confirming the successful encapsulation of the antibiotic within polymer nanoparticles. Four steps of decomposition patterns on drug-loaded chitosan nanoparticles were noticed, as shown in Figure 7. At the beginning, almost 40% weight loss was remarked at 40 °C due to the water molecule release. The next step refers to 75% weight loss at 290 °C, denoting the decomposition of chitosan.

The third step was noticed at 450 °C, with 19% weight loss, possibly due to the decomposition of chitosan and ceftriaxone, respectively. The last step of decomposition was eventually observed at 540 °C of about 10%, due to the decomposition of pure antibiotic.

**Figure 8:**
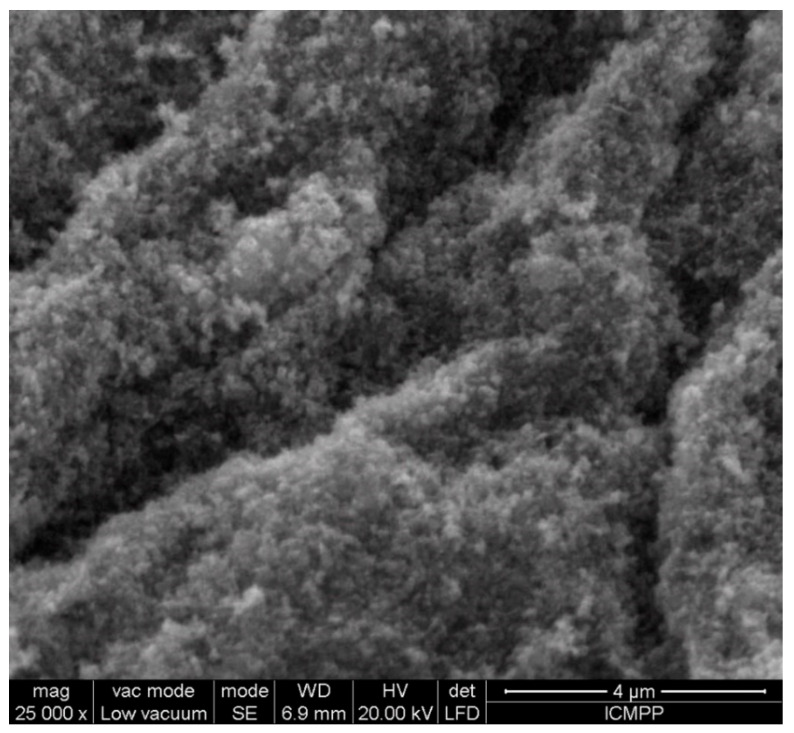
SEM image of ceftriaxone loaded chitosan nanoparticles.

SEM micrographs presented in Figure 8 exhibit the morphological features of ceftriaxone loaded chitosan nanoparticles as being spherical in shape with aggregated particles with approximately 30–250 nm in size.

Small-size particles tend to aggregate due to the raised surface energy. Increased particle size is possibly due to the encapsulation of an antibiotic into chitosan nanoparticles.

## 4. Discussion

In order to design novel formulation-type drug delivery approaches, some specific demands must be respected, especially the increase in therapeutic efficacy and efficiency, minimizing of adverse effects and, most of all, physical and chemical properties, structural and morphological features, biocompatibility and usage in the medical field. The as-prepared carriers based on natural polymers were characterized to check their potential to encapsulate active molecules. Polymer nanoparticles’ capacity to load various antibiotics and then to release the drugs in a controlled and prolonged manner could improve the current therapies. Ceftriaxone-loaded chitosan nano-sized particles present an average particle size of 250 nm and a zeta potential value of 38.5 mV, pointing to the stability of nanoparticles and the high electric surface charge on drug-loaded chitosan nanoparticles. Samples containing an antibiotic-loaded polymer revealed an incipient ceftriaxone release of approximately 15%, and over 2 h of approximately 83%, due to the filling property of chitosan, followed by a decelerated antibiotic release up to 24 h. FTIR spectra of antibiotic-loaded polymer revealed characteristic peaks of chitosan, indicating that it was no alteration of chitosan structure denoting the absence of chemical interaction between ceftriaxone and chitosan nanoparticles. Thermo-gravimetric analysis (TGA) used to analyze the thermal stability of ceftriaxone-loaded chitosan nanoparticles and their weight loss at various temperatures evidenced the successful encapsulation of the antibiotic within polymer nanoparticles. SEM micrographs indicate the morphological characteristics of ceftriaxone-loaded chitosan nanoparticles as being spherical in shape, with agglomerated nano-architectures of approximately 30–250 nm in size. The major aim of this study was to prepare a new nanocomposite based on chitosan and ceftriaxone molecules for targeted drug transport followed by structural and morphological analysis of the obtained nanostructures. The obtained results confirmed that the antibiotic was successfully loaded in the polymer matrix. 

A first step focused on preparation of ceftriaxone encapsulated chitosan nanoparticles and characterization of the obtained samples, then, preceding in vivo evaluation, the antimicrobial effect against multidrug-resistant bacteria and other issues that were not included in this study.

Further studies concern the antimicrobial activity assay that validates the limitation of bacterial multiplication, the improving therapy and the limitation of antibiotic side effects. 

## 5. Conclusions

Multidrug-resistant pathogens represent a major concern worldwide because of the inefficient conventional therapies. In this study, ceftriaxone encapsulated chitosan nanoparticles were prepared and structurally and morphologically tested for possible use in limiting bacterial resistance and improving antibiotic efficacy against some dangerous pathogens. Due to their small size, the obtained nano-hybrids are able to better penetrate the bacterial cell, thus increasing antimicrobial activity by inhibiting gram (+) and gram (−) microorganisms’ multiplication. Our work proves that chitosan nanoparticles could be successfully used in the medical area as a vehicle for antimicrobials, due to their biocompatibility and antibacterial activity, respectively, acting as drug-delivery systems for prolonged and sustained release of the drug. Therefore, nano-antibiotics are novel formulations applied to defeat multi-drug resistant mechanism by inhibiting biofilm formation as well as by enhancing drug efficiency.

## Figures and Tables

**Table 1:** Formulation model for the preparation of ceftriaxone loaded chitosan nanoparticles.

Formulation Code	Amount of Antibiotic (mg/mL)	Amount of Chitosan (mg)	Amount of TPP (mg/mL)	Drug–Polymer Ratio
S1	10	10	0.25	1:1
S2	10	30	0.25	1:3
S3	10	50	0.25	1:5
